# Adolescent health and subsequent risk of self-harm hospitalisation: a 15-year follow-up of the Young-HUNT cohort

**DOI:** 10.1186/s13034-017-0161-8

**Published:** 2017-05-01

**Authors:** Asbjørn Junker, Johan Håkon Bjørngaard, Ottar Bjerkeset

**Affiliations:** 1 Department of Neuroscience, Faculty of Medicine, NTNU-Norwegian University of Science and Technology, Trondheim, Norway; 2 Department of Public Health and General Practice, Faculty of Medicine, NTNU-Norwegian University of Science and Technology, Trondheim, Norway; 3 Forensic Department and Research Centre Brøset, St. Olavs University Hospital, Trondheim, Norway; 4 Faculty of Health Sciences, Nord University, Levanger, Norway

**Keywords:** Self-harm, Hospitalisation, Adolescence

## Abstract

**Background:**

Self-harm is associated with increased suicide risk, and constitutes a major challenge in adolescent mental healthcare. In the current study, we examined the association between different aspects of adolescent health and risk of later self-harm requiring hospital admission.

**Methods:**

We linked baseline information from 13 to 19 year old participants (n = 8965) in the Norwegian Young-HUNT 1 study to patient records of self-harm hospitalisation during 15 years of follow-up. We used Cox regression to estimate risk factor hazard ratios (HR).

**Results:**

Eighty-nine persons (71% female) were admitted to hospital because of self-harm. Intoxication/self-poisoning was the most frequent method (81%). Both mental (anxiety/depression, loneliness, being bullied) and somatic (epilepsy, migraine) health issues were associated with up to fourfold increased risk of self-harm-related hospital admission.

**Conclusions:**

Several health issues during adolescence markedly increased the risk of later self-harm hospitalisation. Current findings should be incorporated in the strive to reduce self-harming and attempted suicides among young people.

## Background

Self-harm behaviours constitute a large health burden, both in terms of health service utilization costs [1], and of increased morbidity and mortality, particularly from suicide [2]. It has been defined as any intentional self-poisoning or self-injury, irrespective of motivation or suicidal intent [3]. The etiology of self-harm is complex [4, 5], and its incidence peaks between 15 and 24 years, occurring most frequently in females [6, 7]. Anxiety and depression are strong risk factors for self-harm behaviour [5, 8]. Further, both internalizing and externalizing disorders and substance use disorders are commonly found comorbidities to self-harm [9]. Also, there is evidence to suggest an association between self-harm risk and drug/alcohol misuse, stressful life events, and socioeconomic disadvantages [5, 8]. Sleep problems have been associated with self-harm in two Norwegian studies [10, 11]. Motivations for self-harm, and associated predictors are overlapping in different sub-populations and the general population, albeit with some differences. For example, among adolescents in the juvenile justice system, externalising disorders and substance use or disorder appear to have limited predictive value [12], perhaps because these conditions are highly prevalent in this population. Patients with psychiatric illness as a group [13], and those with borderline personality disorder in particular [14, 15], are at increased risk of self-harm. There is also emerging evidence of increased risk in relation to autism spectrum disorder in adults [16]. The existing literature has mostly addressed relations between poor mental health and risk of self-harm. Even though associations have been reported between self-harm and physical illnesses such as epilepsy, migraine, asthma, diabetes and eczema [13], evidence is limited—especially among adolescents and young adults. The majority of psychiatric research, and that of North American origin in particular, has used classification criteria from the Diagnostic and Statistical Manual of Mental Disorders (DSM) [17]. Hence, potential associations between physical illnesses and a range of psychiatric disorders including self-harm might not have been as easily studied and identified, since the DSM is a system for psychiatric disorders only, while the ICD system covers all areas of health.

Unfavorable health conditions in adolescence often present with a wide range of physical and psychological symptoms, and their relations to self-harm have often been studied separately. Additionally, few prospective studies have investigated the extent to which risk factors present in adolescence are associated with the self-harm risk in early adulthood. Most studies are also based on self-reported self-harm; limited by non-response and misreporting, and possible underestimation of self-harm prevalence [18].

With the present study, we sought to fill some of these gaps by investigating associations between several dimensions of self-reported physical and mental health symptoms in a community cohort of almost 9000 adolescents, and the risk of self-harm related hospitalisation during 15 years of follow-up. The outcome ascertainment was based on validated outcome data on self-harm recorded from hospital-based patient records.

As previously defined, we investigated self-harm that resulted in hospital admission, without assessing the presence or degree of suicidal intent. However, research indicates that most people whose self-harm leads to hospitalisation carry out the act with at least some degree of suicidal intent [19, 20].

## Methods

### Study population and setting

The Young-HUNT 1 Study [21] was conducted in 1995–97, and all 13–19 year old adolescents (n = 10,202) in Nord-Trøndelag County, Norway, were invited to participate. The Young-HUNT questionnaire was completed by 8983 participants (88%). A total of 114 and 125 questions/items for middle and secondary school, respectively, covered a wide range of aspects of physical and mental health, quality of life, and lifestyle factors (such as alcohol and tobacco use as well as physical activity). Questionnaires were completed during school time, and although non-present invitees on the day of Young-HUNT were invited to take part in the study when they returned, the latter group covered the bulk of non-responders. Two hundred and eighty-five adolescents not attending regular school were mailed the questionnaires at home. Especially trained nurses performed a clinical examination after questionnaire completion.

We excluded 18 persons because their respective Young-HUNT participation date occurred after they experienced their first self-harm hospitalisation (n = 9), or after the date they were registered as lost to follow-up in Nord-Trøndelag County (n = 9).

All participants gave written informed consent for the use of data for medical research (for those <16 years of age, parental consent was also obtained). The study was approved by the Regional Committee for Medical and Health Research Ethics (2010/1924-3).

### Outcome: hospital admissions due to self-harm (1995–2010)

The registration of outcome data has been described in more detail previously [10]. We registered all self-harm episodes that required hospitalisation from 1 January 1995 to 31 December 2010, based on a list of all acute admissions for persons eligible for Young-HUNT 1, at the two hospitals serving the catchment area for the study. The list contained both ICD diagnostic codes and a free text field with the nurses’ description of the reason for admission. We searched this free text field for terms and criteria associated with self-harm ([10], see Appendix). All search-positive patient records were inspected thoroughly, to include or exclude the event as a self-harm episode. Accidental self-harm and events resulting in bodily damage, but without evidence of such an intention, were excluded from further analyses. Examples would include cutting accidents in the kitchen, injuries from sports and games, or the unintended alcohol intoxication at a party. In confirmed self-harm events, we recorded a range of relevant information from patient records, such as date and time for the first self-harm related hospital admission, method(s) used to self-harm, mental and psychiatric comorbidities. The recorded outcome data was merged to the same persons’ baseline data from Young-HUNT by a research technician at HUNT Research Centre, anonymized and returned to the principal investigator (AJ).

### Exposures in Young-HUNT 1 (1995–97)

We included variables from the Young-HUNT 1 questionnaire based on established or proposed associations to self-harm and suicide attempts. Loneliness and being bullied are often accompanied by anxiety and depression, which again may present as psychosomatic symptoms like headache or stomach pain. The burden of various somatic illnesses increases with age, and are known to increase self-harm risk among seniors. We included some somatic illnesses in adolescence to see how this affected the risk of self-harm hospitalisation.

Symptoms of anxiety and depression during the last two weeks were assessed with a five-item version of the Hopkins Symptom Checklist (SCL-5) [22]; “In the last 14 days, have you…”: 1… been constantly afraid and anxious? 2… felt tense or uneasy? 3… felt hopelessness when you think of the future? 4… felt dejected or sad? 5… worried too much about various things? All items had four response options, which we grouped as “not at all/a little” (scored as zero) and “quite a bit/very” (scored as one). We calculated an average scale score ranging from one to four, created a dummy variable where average SCL-5 value >2.00 indicated high mental distress (caseness level), and used single items and the dichotomized SCL-5 variable in the different Cox analyses. Average SCL-5 value variable was analysed for trend measure.

Loneliness was assessed with the item “Do you feel lonely?” Five response options from “very often” to “seldom or never” were dichotomized into “very often/often”, and the remaining options.

Bullying was assessed with one of several items regarding school events, originating from a Norwegian Institute of Public Health survey on child sexual abuse [23]; “Does it happen or has it previously happened at school: you are teased/harassed by other students”. Four response options ranging from “never” to “very often” were dichotomized into “never/sometimes” and “often/very often”.

Two items assessing each of the somatic symptoms stomach pain and headache during the last 12 months (without known medical reason) had four response options that we dichotomized into “never/seldom”, and “sometimes/often” for both symptom items.

Body mass index (weight/height^2^) was calculated from height and weight measurements recorded by a specially trained nurse. We categorized each participant’s body mass index into underweight (BMI <18.5), normal weight (BMI 18.5–25), overweight (BMI 25–30) or obesity (BMI >30), based on international age-and-gender specific cut-off values [24, 25].

Somatic illnesses included five binary variables (yes/no); epilepsy, migraine, asthma, allergy, and ever having had intermittent skin rashes for at least 6 months. The questions assessing asthma, allergy and skin rash were adapted from the ISAAC core questionnaire [26].

Smoking status was categorized as non-smoker/smoker (those who reported daily or occasional cigarette smoking).

Alcohol use was categorized in keeping with previous studies on this cohort [27] as having felt drunk >10 times during their lives, or not/less.

### Covariates

Self-harm incidence differs between age groups and genders. Parental conflict, and an unstable family situation may adversely affect the health and wellbeing in children and adolescents, and increase mental distress. Socioeconomic status is another factor well-known for influencing various aspects and outcomes regarding health. To control for potential confounding by these factors, we used information on age and gender of participants, parental cohabitation situation (whether the participants lived together with non-divorced mother and father, or not) and socioeconomic status (highest educational level for mother or father or both—categorized as primary, intermediate or tertiary. Data on parental educational level were obtained from a national database held by Statistics Norway (SSB) [28], after the end of the follow-up period. Parental educational level refers to parents’ highest education on 1 October the year the offspring turns 16 years old.

### Statistics

With attained age as the time axis, we applied Cox proportional hazard regression analyses, using STATA version 12 for Mac [29]. We performed person-based analyses where the follow-up period covered the time between each participant’s survey completion date and the date when they moved out of the county, died, experienced their first self-harm related hospital admission, or until 31 December 2010—whichever occurred first.

First, we investigated the association of baseline score on (a) caseness symptoms of anxiety and depression (mean SCL-5 score >2.00), (b) each single SCL-5 item, (c) loneliness, (d) bullying, (e) stomach pain, (f) headache, (g) epilepsy, (h) migraine, (i) asthma, (j) allergy, (k) skin rashes, (l) smoking, (m) alcohol use and (n) body mass index with subsequent hospitalisation for self-harm. Each variable was analysed only adjusting for age (as time axis). Analyses were then repeated, adjusted for gender, age, cohabitation situation and socioeconomic status. Hazard ratios (HR) were reported with 95% confidence intervals (95% CI). We used the Schoenfeld residuals test to test the proportional hazard assumption in the Cox analysis [30]. Based on Schoenfeld residuals, we found no indications of violation of this assumption. In order to assess possible reverse causality, we did an additional analysis removing the first 5 months of follow up. We investigated the possibility of statistical interaction between gender and the included health measures, and tested for effect measure modification to see whether an effect measure of a certain variable on self-harm hospitalisation risk was different in males and females. In addition, we examined whether age modified the effect of smoking and alcohol on self-harm risk, by testing for effect measure modification when participants were categorised in middle school (13–16 years old) and secondary school (16–19 years old). Using the STATA add on package—punafcc—[31], we calculated individual population attributable fractions (PAF) for the variables assumed most plausible to have a causal effect on self-harm hospitalisation risk. PAF is an estimate of a specific risk factor’s contribution to the disease burden in a population; how large the reduction in disease or mortality would be if exposure to a risk factor was reduced to a defined, lower level. In the current study setting, PAF would translate to: how many self-harm hospitalisations would be prevented if none of the participants experienced caseness symptoms of anxiety and depression? We chose not to estimate PAFs for factors where direct causality is unlikely (smoking, body mass index), or where an exposure reduction is impossible or difficult to obtain (gender, age, socioeconomic status).

## Results

In this cohort of 8965 adolescents, 4451 (49.7%) were female, and mean age at baseline was 16 years for both genders. Baseline characteristics of the study population are presented in Table 1.

**Table 1:** Descriptive baseline characteristics of the study population

	Total cohort			
	Self-harm		Non-self-harm	
	N (%)	Mean (SD)	N (%)	Mean (SD)
Gender				
Male	26 (29.2)		4488 (50.6)	
Female	63 (70.8)		4388 (49.4)	
Parental socioeconomic status				
Primary education	9 (10.1)		661 (7.5)	
Secondary education	14 (15.7)		2198 (24.8)	
Tertiary education	66 (74.2)		5997 (67.5)	
Missing	0 (0)		20 (0.2)	
Age (in years)		15.9 (1.9)		16.0 (1.8)
Anxiety and depression (SCL-5)				
SCL-5 score		1.9 (0.8)		1.5 (0.5)
Missing	3 (3.4)		174 (2.0)	
Non-caseness symptom level	58 (65.2)		7845 (88.3)	
Caseness symptom level	28 (31.4)		857 (9.7)	
Missing	3 (3.4)		174 (2.0)	
(SCL-5 single items)				
Not felt constantly afraid and anxious	73 (82.0)		8469 (95.4)	
Felt constantly afraid and anxious	13 (14.6)		233 (2.6)	
Missing	3 (3.4)		174 (2.0)	
Not felt tense or uneasy	64 (71.9)		8223 (92.7)	
Felt tense or uneasy	21 (23.6)		445 (5.0)	
Missing	4 (4.5)		208 (2.4)	
Not felt hopelessness thinking of the future	65 (73.0)		7818 (88.1)	
Felt hopelessness thinking of the future	21 (23.6)		877 (9.9)	
Missing	3 (3.4)		181 (2.0)	
Not felt dejected or sad	67 (75.3)		7954 (89.6)	
Felt dejected or sad	19 (21.3)		691 (7.8)	
Missing	3 (3.4)		231 (2.6)	
Not worried too much about things	62 (69.6)		7703 (86.8)	
Worried too much about things	24 (27.0)		981 (11.0)	
Missing	3 (3.4)		192 (2.2)	
Loneliness				
No	68 (76.4)		8227 (92.7)	
Yes	18 (20.2)		509 (5.7)	
Missing	3 (3.4)		140 (1.6)	
Bullied at school				
No	76 (85.4)		8475 (95.5)	
Yes	6 (6.7)		173 (2.0)	
Missing	7 (7.9)		228 (2.5)	
Body mass index (kg/m^2^)^a^				
Underweight (BMI <18.5)	6 (6.7)		401 (4.5)	
Normal-weight (BMI 18.5–25)	59 (66.3)		6485 (73.1)	
Overweight (BMI 25–30)	11 (12.4)		1191 (13.4)	
Obesity (BMI >30)	7 (7.9)		246 (2.8)	
Missing	6 (6.7)		553 (6.2)	
Epilepsy				
No	79 (88.7)		8467 (95.4)	
Yes	3 (3.4)		95 (1.1)	
Missing	7 (7.9)		314 (3.5)	
Migraine				
No	76 (85.4)		8313 (93.7)	
Yes	7 (7.9)		302 (3.4)	
Missing	6 (6.7)		261 (2.9)	
Asthma				
No	77 (86.5)		7691 (86.7)	
Yes	12 (13.5)		1044 (11.8)	
Missing	0 (0)		141 (1.6)	
Allergy				
No	65 (73.0)		5863 (66.1)	
Yes	23 (25.9)		2882 (32.5)	
Missing	1 (1.1)		131 (1.5)	
Skin rash				
No	67 (75.3)		6913 (77.9)	
Yes	21 (23.6)		1785 (20.1)	
Missing	1 (1.1)		178 (2.0)	
Stomach pain				
No	52 (58.4)		6818 (76.8)	
Yes	33 (37.1)		1743 (19.6)	
Missing	4 (4.5)		315 (3.6)	
Headache				
No	31 (34.8)		5186 (58.4)	
Yes	55 (61.8)		3474 (39.2)	
Missing	3 (3.4)		216 (2.4)	
Smoking				
Non-smoker	31 (34.8)		3086 (34.8)	
Smoker (occasionally/daily)	36 (40.5)		1859 (20.9)	
Missing	22 (24.7)		3931 (44.3)	
Alcohol				
Been drunk ≤10 times	45 (50.6)		4669 (52.6)	
Been drunk >10 times	30 (33.7)		2564 (28.9)	
Missing	14 (15.7)		1643 (18.5)	
Total	89		8876	

Results are reported as numbers and percentages [N (%)] except for the continuous variables where mean and standard deviation [Mean (SD)] is reported

*SCL-5* Hopkins Symptom Checklist, 5-item version

^a^Age-and-gender specific body mass index categories based on international cut-off values

Over the follow-up period 3813 participants (42.5%) emigrated out of the study region. Those lost to follow-up in this way were broadly similar to those who remained; e.g. caseness anxiety and depression was 10.2% in those moving and 9.6% in those who remained in Nord-Trøndelag. However, those who migrated tended to have a less well-educated parents (11.7% primary education) compared to those who remained (4.4% primary education).

Average follow-up period was 11.9 years (range 0.02–16.0 years), during this period 89 (1.0%) participants were hospitalised after a self-harm episode in one of the two County Hospitals. Twenty-six (29%) were males, 54 (61%) experienced only one self-harm hospitalisation, and the remaining patients (n = 35) were admitted to hospital after self-harm more than once during follow-up. Mean age at self-harm index episode was 22.6 for males and 20.9 years for females. Self-poisoning (n = 72, 81%) and laceration (n = 13, 15%) were the most frequently used self-harm methods; eight of these patients both cut and intoxicated themselves in the same episode. The estimated incidence rate of hospitalisation for self-harm for the entire follow-up period was 84 per 100,000 person years [95% confidence interval (CI) 67.9–102.8]; 121 per 100,000 person years (95% CI 94.5–154.8) for females, and 48 per 100,000 person years (95% CI 32.5–70.1) for males.

Among the 89 self-harm patients, n = 37 (42%) were under current psychiatric treatment at the time of self-harm index episode. The majority (n = 22) were in an outpatient setting, four were admitted to a psychiatric department, and 11 received combined outpatient and inpatient psychiatric treatment. Most patients (n = 59) had not previously been in contact with psychiatric specialist healthcare.

In the self-harm patients under current psychiatric treatment at index episode, mood (affective) disorders (F30–39 in ICD-10) and neurotic, stress-related and somatoform disorders (F40–49) were equally common, found in over 50% (n = 20). Second most common was disorders of adult personality and behaviour (F60–69, n = 13), followed by mental disorders due to psychoactive substance use (F10–19, n = 11). There were no cases of self-harm (independent of psychiatric treatment status) that had a diagnosis of autism spectrum disorder (F84).

### Mental health measurements and self-harm hospitalisation

As summarized in Table 2, we found several indicators of psychological distress to be strongly associated with increased risk of self-harm. Frequently feeling tense and uneasy, or afraid and anxious, increased the risk of self-harm hospitalisation over four times. Caseness symptoms of anxiety/depression, often feeling lonely, or being bullied, were also associated with more than three times the self-harm risk compared to less symptoms and psychological distress.

**Table 2:** Hazard ratios for self-harm according to indicators of adolescent mental and physical health in the study population (crude and adjusted models)

	No.	Crude^b^	Adjusted^c^
	SH^a^	HR (95% CI)	HR (95% CI)
Gender			
Male	24	1.00	1.00
Female	60	2.55 (1.59–4.10)	2.50 (1.56–4.01)
Parental socioeconomic status			
Primary education	8	1.00	1.00
Secondary education	13	0.47 (0.20–1.14)	0.43 (0.18–1.05)
Tertiary education	63	0.83 (0.40–1.73)	0.73 (0.35–1.53)
Cohabitation status			
Parents live together	51	1.00	1.00
Parents separated/divorced	33	2.59 (1.67–4.01)	2.54 (1.63–3.94)
Anxiety/depression (SCL-5)			
SCL-5 mean score	83	2.98 (2.22–4.00)	2.51 (1.84–3.43)
Non-caseness symptoms anxiety/depression	57	1.00	1.00
Caseness symptoms anxiety/depression	26	4.46 (2.80–7.10)	3.52 (2.18–5.67)
(SCL-5 single items)			
Not felt constantly afraid and anxious	72	1.00	1.00
Felt constantly afraid and anxious	11	5.78 (3.06–10.90)	4.21 (2.21–8.02)
Not felt tense or uneasy	63	1.00	1.00
Felt tense or uneasy	19	5.95 (3.56–9.95)	4.75 (2.82–8.01)
Not felt hopelessness when thinking of the future	64	1.00	1.00
Felt hopelessness when thinking of the future	19	2.88 (1.73–4.81)	2.49 (1.49–4.18)
Not felt dejected or sad	66	1.00	1.00
Felt dejected or sad	17	3.21 (1.88–5.47)	2.46 (1.43–4.25)
Not worried too much about various things	61	1.00	1.00
Worried too much about various things	22	3.01 (1.85–4.89)	2.44 (1.49–4.00)
Loneliness			
Sometimes/less seldom	68	1.00	1.00
Very often/often	16	3.99 (2.32–6.89)	3.31 (1.91–5.73)
Bullied at school			
Never/sometimes	75	1.00	1.00
Very often/often	5	3.39 (1.37–8.38)	3.30 (1.33–8.16)
Body mass index (kg/m^2^)^d^			
Underweight (BMI <18.5)	5	1.47 (0.59–3.67)	1.46 (0.59–3.65)
Normal-weight (BMI 18.5–25)	56	1.00	1.00
Overweight (BMI 25–30)	11	1.06 (0.56–2.03)	0.99 (0.52–1.89)
Obesity (BMI >30)	7	3.15 (1.44–6.92)	3.06 (1.39–6.73)
Epilepsy			
No	75	1.00	1.00
Yes	3	3.82 (1.21–12.13)	3.97 (1.25–12.63)
Migraine			
No	72	1.00	1.00
Yes	7	2.72 (1.25–5.91)	2.34 (1.08–5.10)
Asthma			
No	73	1.00	1.00
Yes	11	1.13 (0.60–2.12)	1.09 (0.58–2.05)
Allergy			
No	62	1.00	1.00
Yes	22	0.73 (0.45–1.19)	0.71 (0.43–1.15)
Skin rash			
No	64	1.00	1.00
Yes	20	1.21 (0.73–1.99)	1.04 (0.63–1.73)
Stomach pain			
Seldom/never	47	1.00	1.00
Sometimes/often	33	2.73 (1.75–4.26)	2.23 (1.42–3.52)
Headache			
Seldom/never	29	1.00	1.00
Sometimes/often	52	2.68 (1.70–4.22)	2.17 (1.36–3.46)
Smoking			
Non-smoker	30	1.00	1.00
Smoker (occasionally/daily)	34	2.01 (1.23–3.29)	1.82 (1.11–2.98)
Alcohol			
Been drunk ≤10 times	45	1.00	1.00
Been drunk >10 times	28	1.30 (0.80–2.10)	1.23 (0.76–2.00)

*SH* self-harm, *HR* hazard ratio, *SCL-5* Hopkins Symptom Checklist, 5-item version

^a^Total number of self-harm patients vary due to complete case analyses with varying number of missing observations

^b^Adjusted for age (as time axis)

^c^Adjusted for age (as time axis), gender, cohabitation situation and socioeconomic status/parental education level at baseline

^d^Age-and-gender specific body mass index categories based on international cut-off values

Adjusted population attributable fractions (PAFs) are presented in Table 3. Caseness symptoms of anxiety and depression PAF was 22.4%, with single item PAFs ranging from 10.1 (often afraid) to 18.3% (often tense/uneasy). Being bullied and feeling lonely was associated with approximately the same risk increase, but due to higher prevalence, loneliness PAF was three times the PAF of being bullied.

**Table 3:** Population attributable fractions for self-harm hospitalisation according to indicators of adolescent mental and physical health in the study population

	No. SH^a^	PAF %^b^ (95% CI)
Anxiety/depression (SCL-5)		
Caseness symptoms anxiety/depression	26	22.4 (18.1–26.6)
(SCL-5 single items)		
Felt constantly afraid and anxious	11	10.1 (8.1–12.1)
Felt tense or uneasy	19	18.3 (15.7–20.8)
Felt hopelessness when thinking of the future	19	13.7 (8.8–18.3)
Felt dejected or sad	17	12.2 (7.5–16.6)
Worried too much about various things	22	15.6 (10.1–20.9)
Loneliness		
Very often/often	16	13.3 (10.1–16.4)
Bullied at school		
Very often/often	5	4.4 (2.6–6.1)
Epilepsy		
Yes	3	2.9 (1.8–4.0)
Migraine		
Yes	7	5.1 (2.1–8.0)
Stomach pain		
Sometimes/often	33	22.8 (13.9–30.8)
Headache		
Sometimes/often	52	34.6 (19.2–47.0)

*SH* self-harm, *SCL-5* Hopkins Symptom Checklist, 5-item version

^a^Total number of self-harm patients vary due to complete case analyses with varying number of missing observations

^b^Adjusted for age (as time axis), gender, cohabitation situation and socioeconomic status/parental education level at baseline

### Physical health problems and self-harm hospitalisation

Diagnosed epilepsy and migraine at baseline increased the self-harm hospitalisation risk almost four, and over two times, respectively. However, these estimates were subject to poor precision due to small number of people in the exposed groups. With regard to psychosomatic symptoms, people reporting frequent stomach pain or headache had twice the risk of self-harm hospitalisation, compared to those experiencing a lesser symptom burden. Stomach pain PAF was 22.8%, and headache PAF was estimated to be 34.6%.

Daily or occasionally smoking was associated with a nearly doubled risk of self-harm hospitalisation. High alcohol consumption resulted in a small risk increase, but the estimate was not precise enough to leave out chance as a possible explanation. Asthma, allergy and skin rashes were not substantially associated with self-harm hospitalisation, neither were underweight or overweight compared to normal-weight. However, obesity increased the risk substantially.

### Sensitivity analysis

Tests for effect measure modification revealed no statistically significant differences between males and females (all interaction p values >0.05). Nor did we find any evidence of statistically significant age differences with regard to smoking or alcohol use (interaction p values 0.519 and 0.775, respectively). After excluding incident cases in the first 5 months of follow-up, results were nearly identical to the main results (Table 4).

**Table 4:** Hazard ratios for self-harm according to indicators of adolescent mental and physical health in the study population (crude and adjusted models)

	No.	Crude^b^	Adjusted^c^
	SH^a^	HR (95% CI)	HR (95% CI)
Gender			
Male	24	1.00	1.00
Female	57	2.42 (1.50–3.90)	2.37 (1.47–3.82)
Parental socioeconomic status			
Primary education	7	1.00	1.00
Secondary education	13	0.54 (0.21–1.34)	0.49 (0.20–1.23)
Tertiary education	61	0.91 (0.42–1.99)	0.80 (0.37–1.76)
Cohabitation status			
Parents live together	49	1.00	1.00
Parents separated/divorced	32	2.61 (1.67–4.08)	2.56 (1.63–4.00)
Anxiety/depression (SCL-5)			
SCL-5 mean score	80	2.80 (2.06–3.81)	2.36 (1.71–3.27)
Non-caseness symptoms anxiety/depression	56	1.00	1.00
Caseness symptoms anxiety/depression	24	4.25 (2.63–6.86)	3.37 (2.06–5.52)
(SCL-5 single items)			
Not felt constantly afraid and anxious	70	1.00	1.00
Felt constantly afraid and anxious	10	5.43 (2.80–10.54)	4.00 (2.04–7.84)
Not felt tense or uneasy	62	1.00	1.00
Felt tense or uneasy	17	5.48 (3.20–9.38)	4.40 (2.55–7.59)
Not felt hopelessness when thinking of the future	63	1.00	1.00
Felt hopelessness when thinking of the future	17	2.65 (1.55–4.53)	2.31 (1.34–3.96)
Not felt dejected or sad	65	1.00	1.00
Felt dejected or sad	15	2.91 (1.55–5.10)	2.25 (1.27–3.99)
Not worried too much about various things	60	1.00	1.00
Worried too much about various things	20	2.80 (1.69–4.65)	2.29 (1.37–3.83)
Loneliness			
Sometimes/less seldom	66	1.00	1.00
Very often/often	15	3.88 (2.22–6.80)	3.24 (1.84–5.70)
Bullied at school			
Never/sometimes	73	1.00	1.00
Very often/often	5	3.45 (1.40–8.55)	3.37 (1.36–8.35)
Body mass index (kg/m^2^)^d^			
Underweight (BMI <18.5)	4	1.22 (0.44–3.36)	1.21 (0.44–3.35)
Normal-weight (BMI 18.5–25)	54	1.00	1.00
Overweight (BMI 25–30)	11	1.10 (0.58–2.11)	1.02 (0.53–1.96)
Obesity (BMI >30)	7	3.28 (1.49–7.22)	3.18 (1.45–7.01)
Epilepsy			
No	72	1.00	1.00
Yes	3	3.99 (1.26–12.68)	4.08 (1.28–12.98)
Migraine			
No	69	1.00	1.00
Yes	7	2.85 (1.31–6.19)	2.45 (1.13–5.35)
Asthma			
No	70	1.00	1.00
Yes	11	1.18 (0.62–2.23)	1.13 (0.60–2.14)
Allergy			
No	60	1.00	1.00
Yes	21	0.73 (0.44–1.20)	0.70 (0.43–1.15)
Skin rash			
No	63	1.00	1.00
Yes	18	1.10 (0.65–1.86)	0.96 (0.57–1.62)
Stomach pain			
Seldom/never	46	1.00	1.00
Sometimes/often	31	2.62 (1.66–4.14)	2.16 (1.36–3.45)
Headache			
Seldom/never	29	1.00	1.00
Sometimes/often	49	2.53 (1.60–4.00)	2.06 (1.29–3.31)
Smoking			
Non-smoker	29	1.00	1.00
Smoker (occasionally/daily)	33	2.08 (1.26–3.43)	1.89 (1.14–3.13)
Alcohol			
Been drunk ≤10 times	45	1.00	1.00
Been drunk >10 times	26	1.28 (0.78–2.10)	1.21 (0.73–2.00)

Excluding self-harm hospitalisations occurring within 5 months after participation in Young-HUNT

*SH* self-harm, *HR* hazard ratio, *SCL-5* Hopkins Symptom Checklist, 5-item version

^a^Total number of self-harm patients vary due to complete case analyses with varying number of missing observations, and start of follow-up postponed 5 months (152 days) from Young-HUNT participation date

^b^Adjusted for age (as time axis)

^c^Adjusted for age (as time axis), gender, cohabitation situation and socioeconomic status/parental education level at baseline

^d^Continuous age- and gender-specific residuals of standard deviation from mean group body mass index

## Discussion

The results from this 15-year follow-up study of 8965 adolescents displayed strong associations between psychological distress and some somatic illnesses and symptoms in adolescence, and subsequent risk of self-harm hospitalisation. Symptoms of anxiety and depression, loneliness and being subject to bullying were all strongly associated with the risk of self-harm hospitalisation. Self-reported stomach pains and headaches were associated with self-harm hospitalisation, as were epilepsy and migraine. Underweight or overweight altered the risk only marginally, but obesity was associated with a substantial risk increase. Smoking and alcohol consumption were also associated with increased risk, yet less than the indicators of mental and physical health. Asthma, allergy and skin rashes were not substantially associated with self-harm hospitalisation.

The incidence rates estimated in our study are lower than might be expected in this age group. A Norwegian study [7] using national patient register data including patients older than 15 years, found an incidence rate for deliberate self-poisonings treated in hospitals at 120 per 100,000 person years, higher among women (144 per 100,000 person years) than men (94 per 100,000 person years). That study was incidence based, which implies that each patient could contribute with repeated hospitalisations, while patients in our study were censored when they experienced their first self-harm related hospitalisation. This could in part explain our lower incidence rates, given that almost 40% of our patients were hospitalized more than once during follow-up, combined with high repetition rates in this patient group [32]. Perhaps more important, Young-HUNT non-participants are as a group presumably at higher risk of self-harm hospitalisation compared to those who participated. In addition, people are lost to follow-up from the date they move out of Nord-Trøndelag county. Over 42% (n = 3813) moved—and were therefore censored—before 31 December 2010, and may have been hospitalised outside our catchment area.

## Strengths and limitations

This is one of the first studies linking a large population-based cohort sample to hospital admissions due to self-harm in adolescents and young adults. The main strengths of this study are the prospective design, long follow-up time, large sample size, and validated clinical outcome measurements, with minimal misclassification. Most previous studies have relied on self-reported self-harm behaviours. Additionally, the Young-HUNT survey makes it possible to investigate and compare the effect of a broad variety of risk factors, among self-harm patients and controls from the same large, representative community population.

There are, however, important limitations to this study. Baseline variables were only measured once, yet some of these might have fluctuated considerably during the 15-year follow-up period. Endpoint-data were registered by four different persons. Based on measures such as introductory training, a guiding algorithm document and discussing difficult cases with the first author, we expect the inter-rater reliability to be acceptable. Nevertheless, no analyses to quantify the exact value were carried out. Further, our analysis was restricted to self-harm hospitalisation, and results cannot be generalized to other and milder forms of self-harm, not leading to hospitalisation. In addition, our study does not explicitly differentiate non-suicidal self-injury (NSSI) from suicidal self-harm (suicide attempts). Previous studies indicate that a high proportion of people admitted to hospital following self-harm have self-harmed with suicidal intent [20].

Moreover, measures of anxiety and depression were based on self-report in this study, which makes direct comparison of results to studies using diagnostic categories difficult. The other measures of symptoms and health conditions were also based on self-report. A diagnostic screening could, therefore, have provided more valid information. However, given the prospective nature of our study, it is likely that possible misclassification would be non-differential. Non-differential misclassification would, with some exceptions for categorical exposures, give more conservative estimates.

Although the study was based on a large sample, self-harm hospitalisation is a rare event and only 89 individuals experienced their first self-harm hospitalisation during follow up, limiting our power to detect small, but potentially clinically important associations. We may have missed some participants, for instance due to moving outside Nord-Trøndelag county while studying, and thereby experiencing their first self-harm hospitalisation in other hospitals. Also, in remote, rural areas of Nord-Trøndelag, people may also have sought primary care or no care at all, rather than travelling large distances to receive hospital care. Nevertheless, the positive prediction value is likely to be high based on the rigorous approach of outcome ascertainment. Additionally, premises for valid PAF estimates includes a causal, non-confounded association with the outcome, and this may not be the case for some or all of the associations investigated.

### Adolescent mental health

Overall, the majority of previous studies report considerably lower rates of mental disorders and psychological distress in those who self-harm and attempt suicide, than in those who die by suicide [33]. Nevertheless, clinical studies of both adolescents [34] and adults [33] who self-harm and present to the emergency department, confirm that around 90% fulfil the criteria of one or more psychiatric disorder(s), and that about 7 out of 10 patients have an affective disorder.

We found a more than 2.5 times increased risk for self-harm with caseness symptoms of anxiety and depression, yet most admissions (65%) occurred among participants with low or normal anxiety and depression scores, which highlights the dilemma of individual versus population-based approach in self-harm and suicide prevention. Calculated population attributable fraction for caseness anxiety/depression was 22.4%, suggesting a noticeable decrease in self-harm hospitalisation numbers if it was possible to reduce mental distress among adolescents to a minimum. Although anxiety and depression often overlap [35], the role of anxiety in suicidal behaviours remain somewhat unclear. In a case–control study of 129 young people presenting with medically serious suicide attempts [34], anxiety disorders occurred in only a seventh of patients. In contrast, results from the prospective, population based Netherlands Mental Health Survey and Incidence Study, lifetime diagnoses of all anxiety disorders (social phobia, simple phobia, generalized anxiety disorder, panic disorder, agoraphobia, obsessive–compulsive disorder) were associated with suicidal ideation and attempts both at baseline and during follow-up [36].

Our findings (also stemming from a population-based study) indicate that symptoms of fear, tension and general anxiety, might be even closer linked to future self-harm risk than common symptoms of lowered mood and depression. In accordance with this observation, follow-up studies of the adult HUNT population showed that while depression alone predicted mortality in nearly all causes of death, only combined anxiety and depression predicted death by suicide [37]. Keeping in mind that the information from single items is limited, it is interesting that positive responses to the SCL-5 items regarding anxiety symptoms displayed larger risk increase than the depression-related ones. This might be explained by the typical age distribution difference, where anxiety often presents at younger age (during teen years) while depression more often begins around 30 years of age [38].

Elevated risk of self-injury among adults with autism spectrum disorder (ASD), compared to those without, has been reported [16]. Unfortunately, we did not have information on ASD symptoms or diagnosis at baseline, and could therefore not estimate the specific association between this disorder and risk of self-harm hospitalisation. Higher rates of self-injury as reported in adolescents and young adults with ASD [39] could arise from using “stereotypical and habitual” self-harm methods: often medically less serious, thus with lower likelihood of presenting to hospital for treatment. Also, the distress following an increasing awareness of social differences and isolation is likely to augment the risk of suicide attempts among adolescents with higher-functioning ASD.

### Loneliness, social and family factors

In a large study of Norwegian teenagers [40], loneliness was associated with suicidal behaviour even when adjusted for use of different intoxicants and familial factors. An Icelandic population-based study of 9th and 10th graders [41] found that breakup with a friend was associated with suicide attempts. Findings from the current study points in a similar direction, with a substantial risk increase for reported loneliness, and an estimated population attributable fraction of 13.3%.

A previous Norwegian population based study [42] indicated that not living with both biological parents, and a diagnosis of any depressive disorder were associated with future self-harm among young and older adolescents. This is in accordance with our results, indicating that not living with both parents (due to separation/divorce) was associated with a more than doubled self-harm risk.

Results from an Australian study following adolescents for 10 years stated that being bullied during childhood increases the risk of self-harm both directly, and indirectly via depression symptoms in early adolescence [43]. Our findings support these results, as we found a more than threefold increased risk of self-harm hospitalisation in those who reported being bullied at baseline. Furthermore, obesity (BMI >30) was associated with substantially increased risk.

### Somatic health and illness

In a US study of children and adolescents with chronic health conditions [44], youth with chronic physical conditions alone (n = 12,554) only had a slight increased risk for self-harm, suicidal ideation and suicide. However, those with co-existing chronic physical and mental conditions, and those with chronic mental conditions had 2–3-fold increased risk. In contrast, another multinational population-based study of 38,000 people [45] linked a wide range of pre-existing physical conditions to suicidal ideation, plans, and attempts. Epilepsy, physical conditions occurring early in life, and increasing number of physical conditions were especially predictive of future suicidality, and adjustment for co-existing mental disorders altered the results only marginally.

We found strong associations between diagnosed epilepsy, migraine and symptomatic headache without known medical reason, and risk of self-harm hospitalisation. This goes along with a British study reporting twice the rate of hospital-presenting self-harm among patients with, compared to people without, epilepsy [46], and a recent Canadian population-based study where migraine headache was prospectively associated with self-harm [47]. In a lifetime perspective, migraine is a common comorbidity to both manic and depressive episodes [48], yet the majority of children and adolescents with migraine do not have a comorbid psychiatric disorder [49]. In addition, recent findings do not suggest a substantial or lasting association between childhood epilepsy and psychiatric disorders and suicidal behaviour [50].

We also found symptomatic abdominal pain to increase the risk of future self-harm hospitalisation, which complies with a review showing that abdominal pain syndromes, both IBS and non-IBS syndromes [51], served as independent predictors for suicidal behaviour.

Estimated PAFs for stomach pain and caseness anxiety and depression were nearly identical. However, an even higher PAF was estimated for headache without known medical reason. Hazard ratios for symptomatic headache and diagnosed migraine were almost the same, but symptomatic headache was much more prevalent. Stomach pain and headache are common and often coexistent somatic symptoms in adolescence, and PAF estimates may not be valid due to confounding. Nonetheless, the high numbers call for possible explanations, of which one could include a bidirectional association between pain symptoms and mental distress caused by various reasons, mediating the disposition to self-harm.

There is evidence suggestive of a link between asthma and suicidal ideation and suicide attempts [52], yet we found no such association in our dataset. This might be explained by ours being a younger study population, with less advanced or serious disease. Similar to asthma, neither skin rashes nor allergy altered the risk substantially.

### Daily smoking and excess alcohol use

In a cross-sectional study of more than 30,000 pupils and students aged 11–19 years [53], results indicated that heavy episodic drinking was associated with doubled risk for self-reported suicide attempt in the last year, and those drinking at a very young age (aged 13 years and younger) were at greatest risk. Our results also indicated that excess alcohol use was associated with elevated risk of future self-harm hospitalisation, similar to results presented in a prospective study of Australian adults [54]. Still, even though our results do indicate an association, we cannot rule out chance as the explaining factor.

Smoking is strongly associated with mental illness, but the causal link has been questioned [55]. Our results indicated an increased risk of self-harm hospitalisation caused by smoking, yet it is likely that this association have arisen due to a strong association between adolescent cigarette smoking and factors predisposing to mental health problems rather than being a causal exposure in itself.

The evidence that smoking and alcohol use act as risk factors for self-harm in our study might be limited, since the prevalence of alcohol and smoking is highly age dependent. However, we did not find support for any evidence of statistical interaction between age and alcohol or age and smoking on subsequent self-harm risk—a result indicating that these behaviours operate as risk factors across adolescence and young adulthood.

## Conclusions

Our results indicated strong associations between several indicators of adolescent health vulnerability and subsequent risk of self-harm hospitalisation. Associations were strongest for indicators of poor mental health, anxiety symptoms in particular. The distribution of self-harm events across the whole symptom score scales for anxiety and depression underlines the need for both individual and population strategies in the prevention and treatment of self-harm behaviour. Most admissions for self-harm occurred among participants reporting low symptom levels on the different analysed factors. This result underscores the limitations of targeted approaches in self-harm and suicide prevention. Though uncertain, epilepsy apparently increased self-harm hospitalisation risk more than caseness anxiety and depression did, and new studies attempting to replicate the results are needed to clarify this further. Additional investigations might provide better understanding of the increased self-harm risk apparently related to headache and stomach pain. Self-harm prevention is a complex matter, as many factors contribute to increased risk. From a population-based point of view, our results indicate that drug administration safety and prescription patterns need further attention. In a clinical setting, extra care should be taken when dealing with young people reporting loneliness or being bullied at school, especially if they also display signs of anxiety or depression, and if they report suicidal thoughts.

## Abbreviations

SCL-5: Hopkins Symptom Checklist, 5-item version
PAF: population attributable fraction
ASD: autism spectrum disorder
SSB: Statistics Norway
